# MapGAN: An Intelligent Generation Model for Network Tile Maps

**DOI:** 10.3390/s20113119

**Published:** 2020-05-31

**Authors:** Jingtao Li, Zhanlong Chen, Xiaozhen Zhao, Lijia Shao

**Affiliations:** 1 School of Geography and Information Engineering, China University of Geosciences, Wuhan 430074, China; duanmu@cug.edu.cn (J.L.); Zhaoxiaozhen@cug.edu.cn (X.Z.); 2 School of Economics and Management, China University of Geosciences, Wuhan 430074, China; addxiyu@cug.edu.cn

**Keywords:** map generation, network tile map, deep generation model, image translation

## Abstract

In recent years, the generative adversarial network (GAN)-based image translation model has achieved great success in image synthesis, image inpainting, image super-resolution, and other tasks. However, the images generated by these models often have problems such as insufficient details and low quality. Especially for the task of map generation, the generated electronic map cannot achieve effects comparable to industrial production in terms of accuracy and aesthetics. This paper proposes a model called Map Generative Adversarial Networks (MapGAN) for generating multitype electronic maps accurately and quickly based on both remote sensing images and render matrices. MapGAN improves the generator architecture of Pix2pixHD and adds a classifier to enhance the model, enabling it to learn the characteristics and style differences of different types of maps. Using the datasets of Google Maps, Baidu maps, and Map World maps, we compare MapGAN with some recent image translation models in the fields of one-to-one map generation and one-to-many domain map generation. The results show that the quality of the electronic maps generated by MapGAN is optimal in terms of both intuitive vision and classic evaluation indicators.

## 1. Introduction

In modern society, the electronic map plays an important role in daily travel navigation, geographic information query, and other services. However, there are still some blind spots in the coverage of electronic maps (such as some remote areas), which to some extent, limits the service level of geographic information data for users and the guidance level for socioeconomic and political purposes. At the same time, the production of electronic maps generally requires vectorization of paper maps first and then involves complex graphic editing manually by industry standards, which consumes a lot of manpower and resources. Inspired by Liao Ke’s work [1], in view of electronic maps from satellite remote sensing images and the powerful image generation capacity of deep generation models, if a generative model can be trained to directly convert remote sensing images into corresponding electronic maps, the production of electronic maps will be accurate and rapid, thereby further improving the service level for society. In fact, many previous researchers have used generative models to solve problems in the remote-sensing community. Ghamisi [2] used conditional generative adversarial nets to generate a digital surface model (DSM) from a single optical image for scenarios in which the elevation information was not available. Zhu [3] designed a 1D generative adversarial network (GAN) and a 3D-GAN for classification of hyperspectral images and achieved competitive results compared to the state-of-the-art methods.

However, using deep generation models to translate remote sensing images to electronic maps involves many difficulties. First, although the image generated by the existing deep generation model obtains good results at a low frequency, the texture is unclear, and the gradient of adjacent pixels is small at a high frequency. Second, electronic maps require continuous roads. However, in remote sensing images, roads may be blocked by other features (such as woodlands and houses) and not shown completely. Network models need to learn to ignore these obstructions and show the blocked part of the road. Third, to produce a standard electronic map, the network model needs to render different elements with correct colors and learn to align the boundaries of some color blocks for the sake of visual aesthetics, which requires the model to have a higher semantic understanding of images.

Some former researchers have experimented with their proposed model on the translation task using remote sensing images and Google maps. However, the purpose of the previous researchers’ map translation task was to prove the feasibility of their proposed model. As long as the generated images were in the style of electronic maps, they were satisfactory because this proved that their model had image translation capabilities, although the quality of generated maps was not high, and it was easy to distinguish between the real electronic maps and the generated electronic maps. For example, Pix2pix [4] established a general framework for image translation based on a CGAN [5]. However, satisfactory results cannot be achieved in specific application scenarios, and the quality of the generated electronic map is poor. A breakthrough of CycleGAN [6] is its ability to solve the problem of image translation in cases where paired training datasets cannot be obtained. When it is applied in the map translation scenario, it is also found that the resulting electronic map has many problems, such as image blurring, unclear texture, and incorrect color rendering. In contrast to the former methods, our method aims to generate multiple domains and more realistic electronic maps at the same time, rather than simply prove the feasibility of the method.

This paper proposes a GAN-based generation model, Map Generative Adversarial Networks (MapGAN), for generating multitype electronic maps from remote sensing images and rendering matrices of special feature elements at the same time. MapGAN improves the generator of Pix2pixHD [7] and adds a classifier to construct map classification loss so as to improve the model’s feature recognition and multitype map generation capabilities. At the end of the study, we compared the electronic maps generated by MapGAN to other models using the same dataset in the fields of one-to-one map generation and one-to-many domain map generation. The experiments show that MapGAN can correctly generate multiple types of maps, and the generated maps perform better in terms of human vision and quality evaluation results, identify a large number of features correctly, and have better color rendering effects in areas such as woodland and water. 

## 2. The Relevant Concepts

### 2.1. Deep Generative Model

A deep generation model is a parametric generation model that uses deep neural network technology to learn the probability distribution of given sample data to generate new data. The deep neural network gives the generative model a stronger learning ability through the complex combination of multilayer perceptrons. In particular, the introduction of convolution technology has greatly improved the ability of the generative model in image processing and image generation. As the predecessor of the deep generation model, the Restricted Boltzmann Machines (RBM) [8] has a relatively simple model architecture consisting of only two layers of networks. Later, models such as the Deep Boltzmann Machines (DBM) [9] and Deep Belief Networks (DBN) [10] emerged as extensions of the RBM. DBNs have achieved great success in image classification and image feature compression. However, it is difficult to calculate the partition function and the prior distribution of these models in the training process, and it is difficult to deal with large data such as images. Variational Auto Encoding (VAE) [11] is a well-known deep generation model developed in recent years after the DBN. It is a differentiable generation model based on encoding and decoding architecture. The model architecture of the encoder and decoder is constructed by the convolutional neural network, which has strong feature extraction and data generation capabilities. Unfortunately, the problem with VAE is that the optimization purpose is to maximize the lower bound of the difference rather than the likelihood function itself; therefore, the generated image is fuzzy and of low quality.

In 2014, Goodfellow [12] proposed a generative adversarial network (GAN) based on game theory. Compared with the previous model, a GAN is the same as VAE in training and can be trained through a differentiable network. A GAN can learn a loss function using a discriminator to optimize the likelihood function itself directly, and there is no difficulty in implementing the partition function; these advantages enable a GAN to generate high-quality data faster and more reliably than other models. However, GANs also have problems such as unstable model training [13] and lack of diversity in generated data. To this end, many researchers have proposed many GAN variants. Among them, CGAN [5] generates data based on the modalities of the input data instead of noise and can control data generation. Deep Convolutional Generative Adversarial Networks (DCGAN) [14] provides a basic framework of a GAN by processing image data through a large number of experiments. StackGAN [15] uses phased pyramid model architecture to achieve the generation of high-resolution bird images from description text. Based on the Wasserstein Generative Adversarial Networks (WGAN) [16], the WGAN with gradient penalty (WGAN-GP) [17] introduces constraints on the discriminator in the form of regularization, which greatly improves the stability of training. In one study, Least Squares Generative Adversarial Networks (LSGAN) [18] changed the original GAN’s objective function from cross-entropy loss to least squares loss, which effectively improved the model’s training speed and the quality of the generated images. In recent years, these generative models of the GAN series have shown amazing results in the fields of image generation [19], text-to-image synthesis [20], image translation [4], and image super-resolution [21].

### 2.2. Image Translation

Image translation refers to the processing of the images in domain A to output the corresponding images in domain B. Many problems in the field of computer vision can be attributed to image translation. For example, the problem of image super-resolution can be seen as the translation from low-resolution images to high-resolution images, and image colorization can be seen as the translation from single-channel grayscale images to multichannel color images. There are two ways to implement image translation: supervised learning and unsupervised learning. Given two edge probability distributions, there are infinite kinds of joint probability distributions that meet the requirements, so the unsupervised image translation problem is relatively difficult to implement. Nevertheless, since it is easier to find a single type of dataset in practice, the application of unsupervised image translation models is more convenient.

Although convolutional neural networks have achieved amazing results in solving image translation tasks of painted art, there are still problems such as the low quality of generated images and complex loss function design. Until the emergence of GANs, a new method was sought for the task of image translation. The GAN solved the problems of the convolutional neural network by training a discriminator to learn the loss function automatically, and this generated higher quality images. 

In recent years, many image translation models have been developed based on GANs. Based on the CGAN, Pix2pix’s [4] generator generates data from the given image. This work is the basis of many subsequent GAN-based image translation models. CycleGAN [6] consists of two generators and two discriminators and adds a cycle constraint to the original GAN’s loss function, which solves the problem of training image translation models without paired datasets. The VAE + GAN [22] model further solves this problem by using shared latent space. Pix2pixHD [7] can generate high-resolution images of 2048 * 1024 from semantic label maps using two generators: global generator network and local enhancer network. TextureGAN [23] achieves fine-grained texture control in deep image synthesis by introducing local texture loss and local content loss. However, the above models can transfer the styles only between two domains at a time, which is inefficient in tasks that aim to generate multiple domain images. In StarGAN [24], however, a multidomain translation network was proposed using a single generator, and this method is only applied when there is no feature mismatch between domains such as face attribute modification. Later, Semantic Consistency and Identity Mapping Multi-Component Generative Adversarial Network (SC-IMGAN) [25] also provided a multi-component one-to-many translation model to address the Person re-identification (Re-ID) problem using semantic consistency and identity mapping. Rahman [26] also successfully applied the image translation model to the domain generalization task and achieved state-of-the-art performance. 

Most image translation models are used to solve specific problems. For some universal translation models, such as Pix2pix and CycleGAN, when applied to map generation tasks, the generated electronic maps cannot accurately identify and render elements such as woodland, waters, and roads. At the same time, there are problems such as blurred texture and low quality. In contrast, MapGAN builds the model from the specific scene of the electronic map generation and uses some targeted optimization measures to improve the accuracy and aesthetics of the generated maps.

## 3. Methodology

In this section, we first discuss the reason for and method of using auxiliary information called render matrices to control color rendering in the task of generating electronic maps, then we describe our proposed MapGAN, a framework to generate multitype electronic maps based on remote sensing images and a render matrix.

### 3.1. Render Matrix

**Figure 1:**
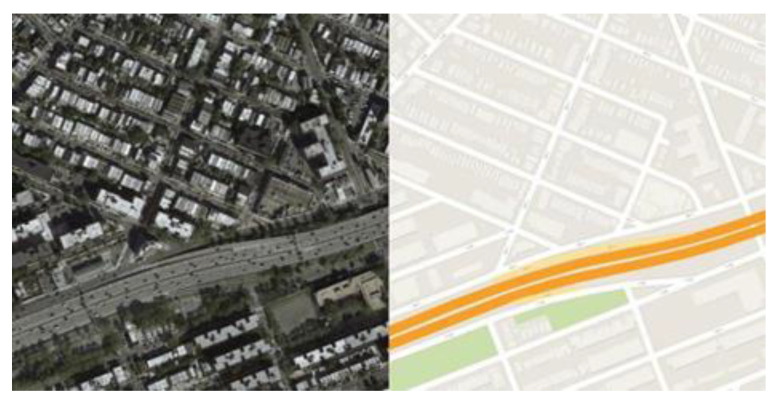
A pair of training data that render the green space as a square for aesthetic reasons.

In each type of electronic map, different colors represent different types of features. Whether the color rendering is correct is an important aspect for evaluation of the quality of the generated electronic map. However, there are many difficulties for the model in learning how to correctly render the generated electronic map, as shown below: (1) Standard color rendering of an electronic map needs to refer to various aspects of the map’s attribute information. For example, the highway is rendered as orange based on the geographic feature information extracted from the geographic entity database. Unfortunately, such attribute information cannot be extracted from remote sensing images by neural networks. (2) The model needs to consider aesthetics when rendering the generated electronic map colors. For example, sometimes to improve the aesthetics of the map, the standard electronic map production process renders green spaces into standard squares, although this may include some nongreen space components, as shown in Figure 1.

**Figure 2:**
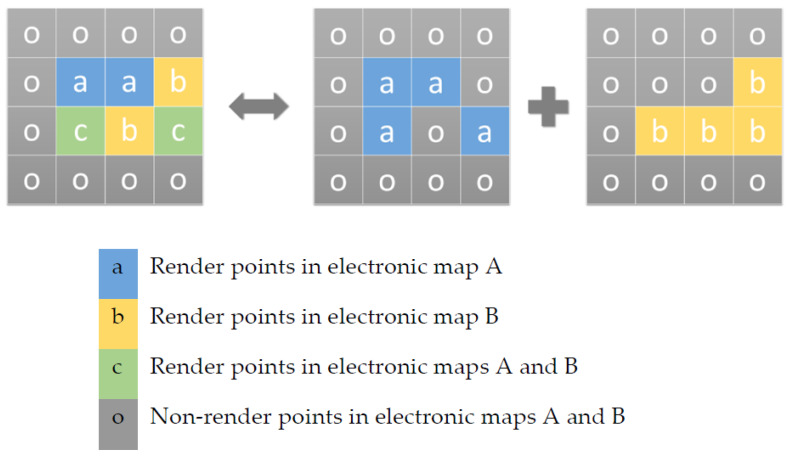
An example of using a rendering matrix of high-speed road features in a scenario where two types of electronic maps, map A and map B, are generated. The left side of the equivalence symbol is the render matrix, which uses different letters to encode the rendering information of the highways in maps A and B. The right side of the equivalence symbol is the equivalent expression of the rendering matrix, which reflects the rendering information of the highways in maps A and B obtained from the rendering matrix.

To address this problem, we used auxiliary information called render matrices to control the color rendering that needs to consider the properties of geographical entities and aesthetics. A render matrix is a two-dimensional matrix with the same width and height as the remote sensing image in the model input. Each render matrix contains the rendering information of a certain feature in various electronic maps to be generated, and this information is encoded and stored in each matrix element. The rendering information stored by the matrix elements is divided into three categories: Category 1 represents that the corresponding position in all types of generated electronic maps does not need to render this type of feature. Category 2 represents that the corresponding position in a certain type of electronic map needs to render this type of feature. Category 3 represents that the corresponding position in several types of electronic maps needs to render this type of feature. For example, when generating the two types of electronic maps, map A and map B, based on a remote sensing image, the correct color rendering of high-speed roads needs to refer to the third-party road network information, which cannot be directly obtained from the remote sensing image, so we used a render matrix as auxiliary information in the model input. The specific coding and meaning of the matrix element are shown in Figure 2.

### 3.2. Map Generation Adversarial Networks

**Figure 3:**
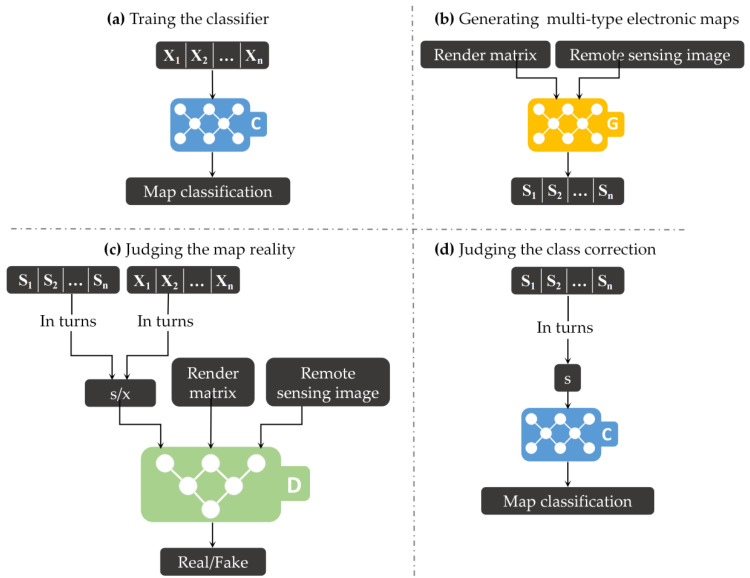
The overall architecture of Map Generative Adversarial Networks (MapGAN), consisting of three modules: a discriminator, D, a generator, G, and a classifier, C. (**a**) We trained classifier C to have map classification capabilities using n types of real electronic maps. (**b**) G outputs the generated n type electronic maps based on remote sensing images and the render matrix, (**c**) D tries to distinguish between real and generated maps corresponding to remote sensing images and the render matrix. (**d**) C tries to judge the category of each generated map.

Our goal was to train the generator G that learns the mapping from the remote sensing image y and the rendering matrix R to various types of electronic maps S, where $S=\{S_{1},S_{2},\ldots ,S_{n}\}$ is used to denote the generated n-type electronic map collection. G simultaneously outputs multitype electronic maps corresponding to y, and we used y, R, and one type of electronic map as inputs to the discriminator to perform image reality discrimination. We also introduced a classifier to determine whether the electronic map generated in S belongs to the correct category. That is, MapGAN evaluated the generated electronic map from the reality and the correctness of the category, with the hope that MapGAN could learn the correct meaning of each element in the rendering matrix and the differences between different types of electronic maps. Supposing we use $X=\{X_{1},X_{2},\ldots ,X_{n}\}$ to denote the n-type target electronic map collection corresponding to S, the training process of our proposed approach is illustrated in Figure 3.

To help MapGAN to learn the meaning of the render matrix and the characteristics and differences of different types of electronic maps more quickly and efficiently, we proposed the following loss terms to form the final loss function of MapGAN:

**Feature Render Loss.** Note that we can use a binary graph $B_{j}^{i}$ to indicate the render information of a feature element i in a certain type of electronic map j, and a pixel with a value of 1 indicates that the position is the corresponding feature. Hence, the render matrix can be seen as a combination of several binary graphs. We can also obtain the binary graph $R_{j}^{i}$ corresponding to $B_{j}^{i}$ from the render matrix. To further promote the model’s use of the render matrix to achieve the correct rendering of corresponding features in different types of electronic maps, we adopted a feature render loss as follows using a binary graph.

(1) $$L_{frl}=\lambda_{1}\sum_{i}\sum_{j}\frac{1}{n_{j}^{i}}\left(\left||R_{j}^{i}-B_{j}^{i}|\right|\right)$$

In Formula (1), $R_{j}^{i}$ and $B_{j}^{i}$ both represent the extracted binary graph of element i in the class j electronic map but from the render matrix and generated electronic map of class j. $n_{j}^{i}$ represents the sum of pixels in $R_{j}^{i}$, the pixel value of which is 1. $\lambda_{1}$ is the weight of the loss term.

**Map Classification Loss.** The discriminator of the generated network can only guarantee the reality of the generated image; that is, it can only ensure that the generated image is an electronic map, but in the task of generating multiple types of electronic maps, the category accuracy of the generated electronic map cannot be guaranteed. To address this problem, MapGAN judges the type of the generated map by adding a pretrained map classifier C. We constructed the cross entropy loss term to help MapGAN generate multiple correct types of maps.

(2) $$L_{mcl}=\lambda_{2}\sum_{s\in S,x\in X}C(x)\log\frac{C(x)}{C(s)}$$

In Formula (2), C(x) and C(s) represent the output of the classifier C with x and s as input, respectively, both of which are n-dimensional probability distribution vectors, the same as S and X. It should be noted that we used the output probability distribution of the target electronic map as the real distribution corresponding to the generated electronic map because we believe that when the training accuracy of the classifier C reaches the peak, compared with one-hot vectors, the output probability of the target electronic map is more representative of the real probability distribution.

**AdaptivePerceptualLoss.** The map generation discussed in this paper can be seen as a special style transfer. In this study, the content image was the remote sensing image, and the style image was the electronic map. The purpose of map generation was to retain certain content of the remote sensing image and convert it into the network electronic map style image. Gatys [27] designed the loss function during the training of the style transfer model as two terms, one to measure the content similarity between the input content image and the output composite image, and the other to measure the style similarity between the input style image and the output composite image based on the Gram matrix. We made the following improvements based on Gatys’ study: (1) We added a feature loss term composed of the features extracted by the discriminator. Xian [23] used similar loss terms in their work and proved that this can stabilize the training of the generator to output more realistic images. (2) A priori test was used to select the network layer in the model proposed by Visual Geometry Group (VGG-19) for feature loss establishment. The mathematical formula of the adaptive perceptual loss $L_{p}$ is as follows:

(3) $$L_{p}=L_{fd}+L_{fg}+L_{s}$$

In Formula (3), $L_{fd}$, $L_{fg}$, and $L_{s}$ represent the feature loss of the discriminator, the feature loss of the VGG-19 model, and the style loss of the VGG-19 model, respectively. The mathematical expression of each loss term is as follows:

(4) $$L_{fd}=\lambda_{3}\sum_{s\in S,x\in X}\sum_{t}\frac{1}{n_{t}}{\left(D_{t}(s,R,y)-D_{t}(x,R,y)\right)}^{2}$$

(5) $$L_{fg}=\lambda_{4}\sum_{s\in S,x\in X}\sum_{t}\frac{1}{n_{t}}{\left(V_{t}(s)-V_{t}(x)\right)}^{2}$$

(6) $$L_{s}=\lambda_{5}\sum_{s\in S,x\in X}{\left(Gram(s)-Gram(x)\right)}^{2}$$

In Formulas (4), (5), and (6), $\lambda_{3}$, $\lambda_{4}$ and $\lambda_{5}$ represent the weights of each loss term. $n_{t}$ represents the total number of features in the t-th network layer, $D_{t}(s,R,y)$ represents the features extracted by the discriminator with the input of the generated electronic map s, render matrix R, and the remote sensing image y at the t-th layer. V represents the VGG-19 model. The style loss term uses the Gram matrix to express the image style, and the Gram matrix is defined as the sum of the inner products of any two feature matrices in a specific network layer. The meanings of the remaining variables are the same as the previous formulas. 

**Adaptive Adversarial Loss.** Adversarial training is widely used in image translation models. During the training process, the discriminator is used to automatically learn the loss function to guide the generator to generate more realistic images. However, Mao [18] pointed out that there is a problem with the original adversarial loss function of GAN: when the generated fake samples are far away from the decision boundary, but still on the real sample side, using the original sigmoid cross-entropy loss function to update the generator causes the vanishing gradient problem. Therefore, we used the proposed least squares loss function to solve the premature disappearance of the gradient and combined the classic L1 loss function to further improve the stability of MapGAN training. The mathematical formula of the adaptive adversarial loss function is as follows:

(7) $$L_{adv}(D)=\lambda_{6}\{\frac{1}{2}E_{y∼p_{y}(y)}[{\left(D(G(y,R),R,y)\right)}^{2}]+\frac{1}{2}E_{x∼p_{x}(x)}[{\left(D(x,R,y)-1\right)}^{2}]\}$$

(8) $$L_{adv}(G)=\lambda_{7}L_{1}+\lambda_{6}\frac{1}{2}E_{y∼p_{y}(y)}[{\left(D(G(y,R),R,y)-1\right)}^{2}]$$

In Formulas (7) and (8), $L_{adv}(D)$ and $L_{adv}(G)$ represent the adaptive adversarial loss function of the discriminator and generator, respectively, and $\lambda_{6}$ and $\lambda_{7}$ represent the weights of the corresponding loss terms. The meanings of the remaining variables are the same as the previous formulas. 

**Full Objective.** Combining the above four loss terms, the objective functions to optimize G and D are written, respectively, as:

(9) $$\begin{array}{l} L(D)=\min L_{adv}(D)\\ L(G)=\min\left(L_{frl}+L_{mcl}+L_{p}+L_{adv}(G)\right) \end{array}$$

where the loss function of the discriminator only includes the corresponding adaptive adversarial loss term and the loss function of the generator includes all the above loss terms.

### 3.3. Map Generation Adversarial Networks

**Figure 4:**
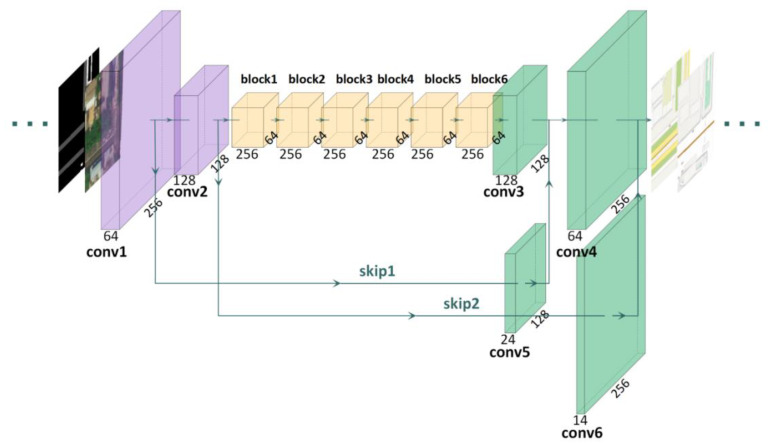
The generator architecture of MapGAN.

**Generator.** The generator architecture of the MapGAN model is based on the network model used in Pix2pixHD. It consists of three parts: the downsampling layer, the residual block [28], and the upsampling layer, as shown in Figure 4 (where purple represents the downsampling layer, pink represents the residual block, and cyan represents the upsampling layer). The improved aspects of the generator include three aspects: (1) rather than 7 × 7 convolution kernels, the maximum kernel size was set to 5 × 5. In practice, three 3 × 3 convolution kernels replaced one 7 × 7 convolution kernel. The purpose is to enhance the model’s sensitivity to the details of each image patch and reduce the training parameters by reducing the convolution kernel receptive field. (2) Six residual blocks were added, with each block consisting of two convolutional layers. The added residual block can improve the model performance by appropriately increasing the network depth without generating any gradient propagation problem. (3) Two skip connections were added between the downsampling layer and the upsampling layer to achieve cross-layer information transfer, effectively helping the model to reuse the features extracted by the downsampling layer when constructing the electronic map during the upsampling stage. 

**Figure 5:**
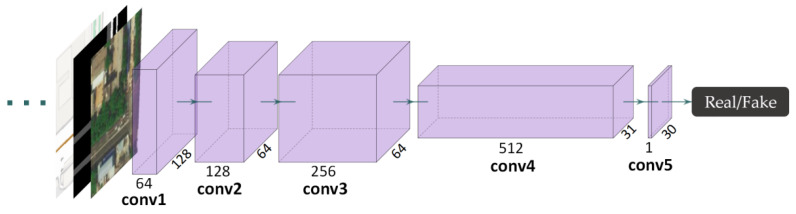
The discriminator architecture of MapGAN.

**Discriminator.** Adapted from Pix2pix [4], the discriminator of the MapGAN model uses the receptive field at a size of 70 × 70 to judge image patches and outputs a matrix representing the discrimination results of each image patch. We also added a convolutional layer to process the output matrix to get the final true probability, instead of simply averaging it. The specific architecture diagram is shown in Figure 5. In practice, the input of patchGAN contains not only the remote sensing image and the electronic map but also all the render matrices in the generator input.

**Classifier.** In this study, we used the Xception model as our classifier, which is an improvement on the Inception v3 model. It uses depthwise separable convolution to replace the replacement convolution operation, thereby improving the model’s effect without increasing the network complexity.

## 4. Results and Discussion

We first tested MapGAN in the one-to-one domain map generation scenario of generating Google electronic maps from Google remote sensing images. In this section, we also show the empirical results of MapGAN in the one-to-many domain map generation scenario that generates Baidu and Map World electronic maps from Baidu remote sensing images at the same time. In both map generation experiments, we conducted relevant ablation studies and compared results with the state-of-art model in terms of visual and quantitative evaluation.

### 4.1. Baseline Models

As our baseline models, we used Pix2pix [4], a mode seeking generative adversarial network (MSGAN) [29] and BicycleGAN for comparison with MapGAN in the one-to-one domain map generation experiment and StarGAN in the one-to-many domain map generation experiment.

Pix2pix is a general-purpose image translation architecture based on conditional adversarial networks that is trained by supervised learning. Since Pix2pix was proposed, a large number of improved image translation models have been proposed, However, due to the versatility and canonicity of Pix2pix, we still used it as a baseline model in this study.

An MSGAN, mode seeking generative adversarial network is equivalent to adding a mode seeking regularization term based on the CGAN to improve the stability and diversity of model training.

BicycleGAN is a one-to-many domain image translation model. BicycleGAN’s innovation lies in ensuring the bijective consistency between the latent code and the output mode, thus preventing the problem of mode collapse and generating better results.

StarGAN is a novel model that can perform image-to-image translations for multiple domains using only a single model. By using a domain label and mask vector, StarGAN allows simultaneous training of multiple datasets with different domains within a single network, which leads to StarGAN’s superior quality of translated images compared to existing models.

### 4.2. Datasets

The Google Maps dataset was made from satellite and electronic map tiles from Google maps, with image sizes of 600 × 600 pixels [4]. The training set and the test set contained 1096 and 1042 pairs of remote sensing-electronic map images, respectively. We used this database in the one-to-one domain map generation experiment.

**Figure 6:**
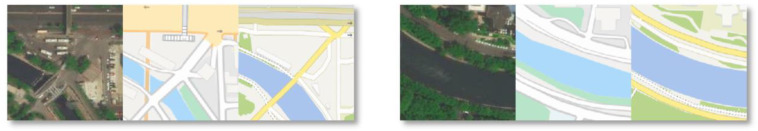
Two training samples in the dataset we made for the one-to-many domain map generation task. Each training sample contains three images; from left to right are the remote sensing image, Baidu map, and Map World map.

The Baidu and Map World Maps dataset was made by downloading Baidu and Map World electronic maps and remote sensing images of the corresponding areas using BIGE MAP software. The size of each image was 256 × 256 pixels. The training set contained 1500 training samples, and the test set contained 500 training samples. Each training sample contained a remote sensing image and a Baidu and Map World electronic map of the same area. We used this database in the one-to-many domain map generation experiment. Two sample examples are shown in Figure 6.

### 4.3. Evaluation Metrics 

Given the unimodal nature of the target translation function in the task of generating electronic maps, we mainly used the average pixel translation accuracy to evaluate the performance of the model in map translation tasks. For a single pixel, if the difference in pixel values is less than 16, the translation result of the pixel is correct; otherwise, the translation result of the pixel is incorrect. The percentage of correctly translated pixels in a remote sensing image is the accuracy of the model in a map translation task. To evaluate the image quality of model generation comprehensively, we used Kernel Maximum Mean Discrepancy (Kernel MMD), Fréchet Inception Distance (FID) [30], mode score, and inception score evaluation indicators, extracting the feature distribution of the target image and the generated image on the penultimate layer and mapping them into Gaussian random variables. In practice, Kernel MMD was calculated using the pretrained resnet34 model on the ImageNet dataset, and the remaining indicators were calculated using the pretrained Inception v3 model on the ImageNet dataset. When using the FID indicator, we used the simplified Frechet distance [31] between two distributions to represent the degree of realism of the generated image.

### 4.4. Training Details

MapGAN used some of the model settings recommended by the DCGAN [14]. The output layer of the generator used the tanh activation function, and the remaining layers used the Relu activation function. MapGAN used the global average pooling layer instead of the full connection layer and used the convolution technique instead of the max-pooling operation. The discriminator used the LeakyReLu activation function with a slope of 0.2. During the model training phase, an Adam optimizer [32] with momentum terms of 0.5 and 0.999 was selected to train a total of 200 epochs. The learning rate was set at 0.002 for the first 100 epochs and decreased linearly to 0 for the last 100 epochs. Training samples for each iteration were randomly obtained from the dataset. The dropout layer with an activation probability of 0.5 was added to the hidden layer of the generator. At the same time, several groups of experiments were set to determine the optimal weight range of each loss item. The third experiment of Section 4.3 shows part of the exploration process. The final weight of each loss term is $\lambda_{1}=\lambda_{2}=\lambda_{7}=100$, $\lambda_{3}=10$, $\lambda_{4}=0.1$, $\lambda_{5}=1e+5$, and $\lambda_{6}=1$. We also used data enhancement techniques to improve the learning ability and robustness of the model, including image rotation and cropping operations.

The experiment was carried out on a workstation with 1 NVIDIA M40 GPU, 4 Inter Xeon Platinum 8163@2.5ghz CPUs, and 30 GB of RAM. It took about three hours to train the model in our one-to-one domain map generation task and five hours in the one-to-many domain map generation task.

### 4.5. One-to-One Domain Map Generation

In this experiment, since our goal was to generate only one kind of electronic map, the classifier was not used in the training process, and we expressed each render matrix as a binary map. The features represented in the binary map were water, woodland, and highway roads.

#### 4.5.1. Feature Loss Term Construction Analysis

To construct the feature loss term more accurately, we explored and selected the reasonable feature extraction network layer when constructing the $L_{fg}$ loss term. From a macro perspective, the shallow network in VGG-19 extracted texture detail features. As the depth of the network grew, the features extracted by the model became more high-level and abstract. From this, we could judge that a reasonable feature comparison layer should be concentrated in the shallow layer. Therefore, we extracted the feature matrices of Relu1_1 to Relu3_4 in the VGG-19 model of the target electronic map and remote sensing images and calculated the L2 loss of the two feature matrices in each layer. The final calculation result of each layer was the average result of 100 pairs of data samples tested in this layer. The experimental results are shown in Table 1. The results show that the L2 loss value of the Relu1_1 layer was the smallest. Therefore, the Relu1_1 layer was finally selected to calculate the $L_{fg}$ loss term.

#### 4.5.2. Weight Analysis of Loss Term

**Figure 7:**
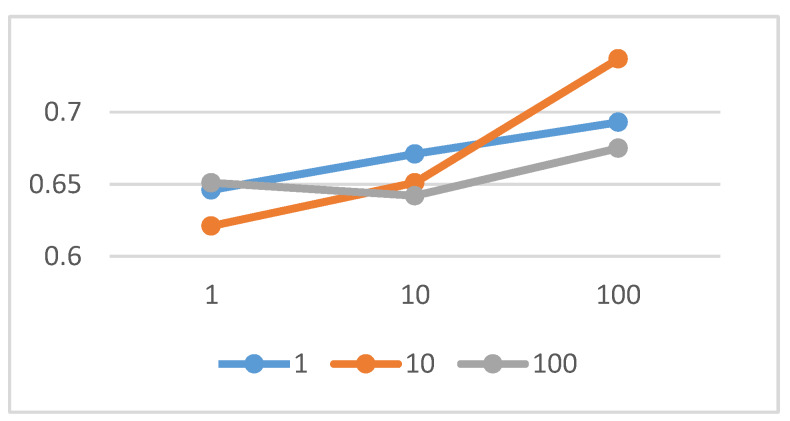
Test of the effect of different values of $\lambda_{17}$ and $\lambda_{3}$ on model performance.

We explored the impact of different weight settings of loss terms on the accuracy of the model map translation. For the convenience of description, the symbol $\lambda_{17}(\lambda_{17}=\lambda_{1}=\lambda_{7})$ was introduced. Under the weight settings of 1, 10, and 100, the experiment tested the influence of $\lambda_{3}$ and $\lambda_{17}$ on the accuracy of MapGAN map translation. The experimental results are shown in Figure 7. The results showed that the larger the value of $\lambda_{17}$ set in the range of 1 to 100, the better the model effect would be. Meanwhile, when $\lambda_{3}$ was in the order of 10, the model training effect would be better than the other two orders of magnitude. Therefore, the parameters finally selected in this study were $\lambda_{17}=100$, $\lambda_{3}=100$.

#### 4.5.3. Control Analysis of Binary Graph Channel Influence on the Model

**Figure 8:**
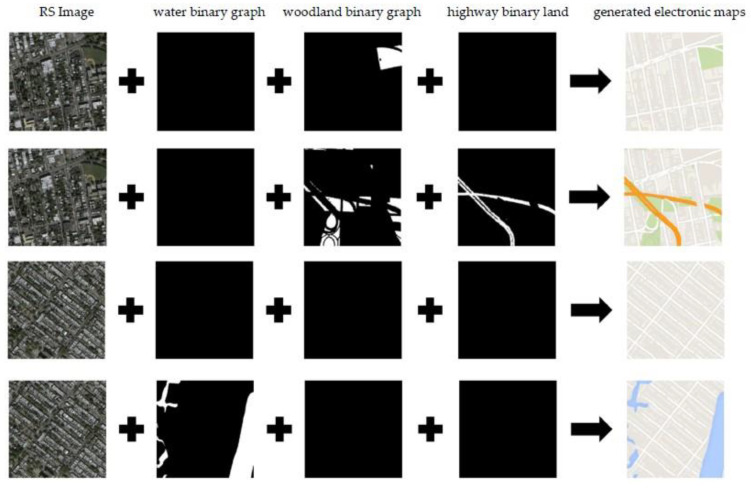
Test for the influence of three binary graph channels in model input on color rendering of the generated electronic map. RS image: remote sensing image. The middle three columns are binary maps corresponding to different elements.

We investigated the influence of three binary graph channels in the model input on the color rendering of the generated electronic map. The three binary graph channels were woodland area binary graph, water land area binary graph, and highway area binary graph. In the formal training of MapGAN, the binary graphs of three channels corresponded to the remote sensing images one by one; however, this experiment broke this correspondence and used a random combination to construct the model input to explore the influence of three binary graph channels on the color rendering of the generated electronic map. The results are shown in Figure 8. The results showed that the woodlands, water, and highways in the electronic map were essentially the same as the corresponding binary graph in shape, size, and location and were drawn evenly. Each binary graph channel had complete control over the generation of corresponding elements in the electronic map.

#### 4.5.4. Qualitative Evaluation

**Figure 9:**
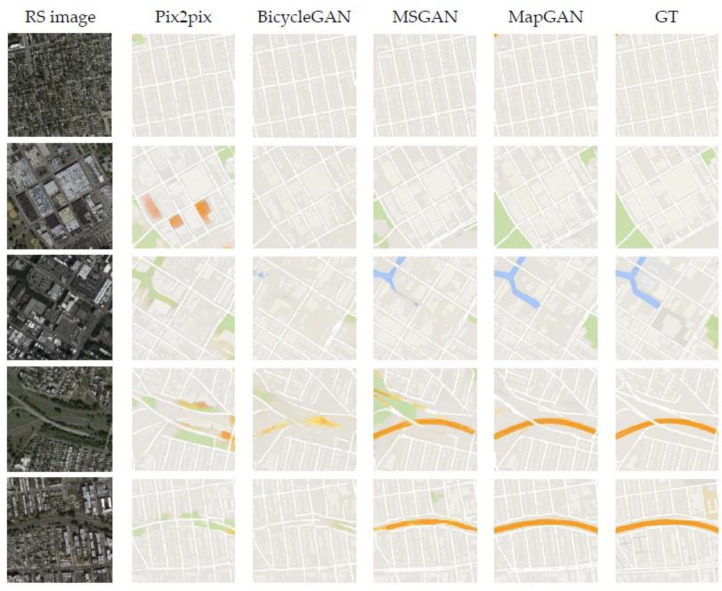
Comparison results of MapGAN and some other image translation models in the one-to-one domain map generation experiment to generate Google maps. The images from left to right are remote sensing images and the Google maps generated by MapGAN, Pix2pix, BicycleGAN, the MSGAN, and MapGAN, the real Google maps.

We used the optimal MapGAN model developed by a large number of experiments to generate tile maps in this experiment and compared the quality of generated maps with some recent image translation models using the same test set. Figure 9 shows the comparison results. We observed that our method provides a higher visual quality of map generation results on the test dataset compared to the other models. The most obvious comparison is the rendering of feature colors, which is because MapGAN refers to the render matrix rather than relying on the extracted unreliable features to identify and render features. Although we did not take targeted optimization measures, MapGAN has an advantage in the accuracy of generating roads for the identification of residential blocks. One possible reason is that the model’s adaptive perceptual loss pays more attention to low-dimensional rather than high-dimensional features.

#### 4.5.5. Quantitative Evaluation

Table 2 shows the results of MapGAN and other image translation models in this one-to-one map generation task over the entire test set under different evaluation indicators. Pix2pix has the worst overall performance. The MSGAN only scores lower than BicycleGAN by 0.020 under the inception score evaluation, and the rest of the scores are relatively high. MapGAN’s overall performance is stronger than the MSGAN and performs relatively worse only under FID evaluation. We find that no model can perform optimally under all indicators. If we sort the models according to their overall performance, the result is MapGAN > MSGAN > BicycleGAN > Pix2pix, which is consistent with the visual performance in Figure 9. Therefore, MapGAN performed best under both quality and numerical evaluation in this experiment.

#### 4.5.6. Ablation Study

**Figure 10:**
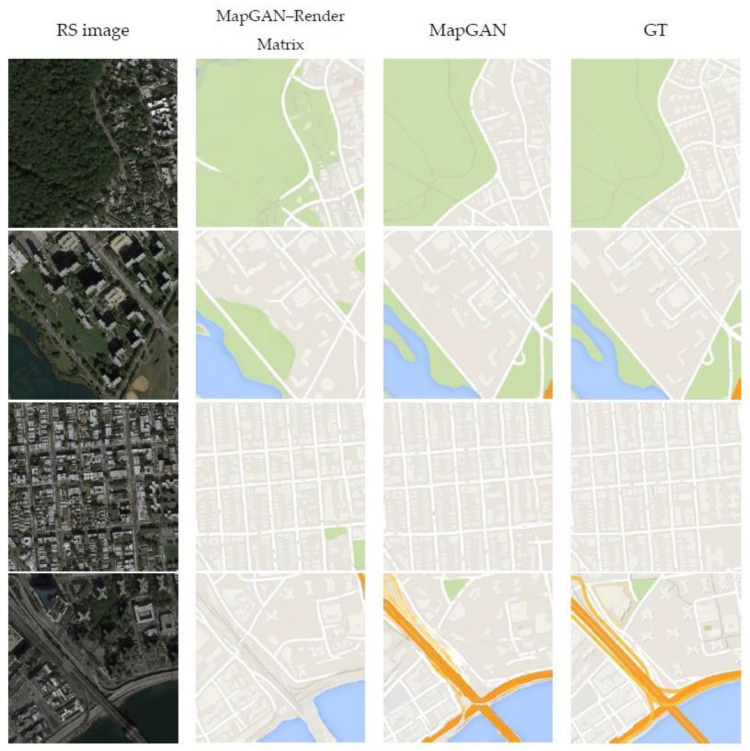
Comparison results of the map generated by MapGAN before and after the removal of the render matrix in the one-to-one map generation experiment. The images from left to right are remote sensing images, the Google maps generated by MapGAN without using render matrix, the Google maps generated by MapGAN, and the real Google maps.

To test the effect of the render matrix on the model generation effect, we removed the render matrix from the model input and evaluated it again. Figure 10 shows the comparison results of the map generated by the model after the removal of the render matrix. It shows that except for the features represented by the render matrix that cannot be correctly color rendered, the effect of generating other elements is not much different after the removal. The last row of Table 2 shows that the model without the input of the render matrix is slightly worse than the MSGAN under mode score and FID evaluation, but the comprehensive evaluation result is still optimal. This proves that our improvements in the model architecture and loss function are effective, and the role of the render matrix is only used to provide color rendering information when the corresponding features are generated.

### 4.6. One-to-Many Domain Map Generation

In this experiment, we used the same parameter settings as in Section 4.5. The model input contained three render matrices, and the corresponding features were water, woodland, and highway roads.

#### 4.6.1. Qualitative Evaluation

**Figure 11:**
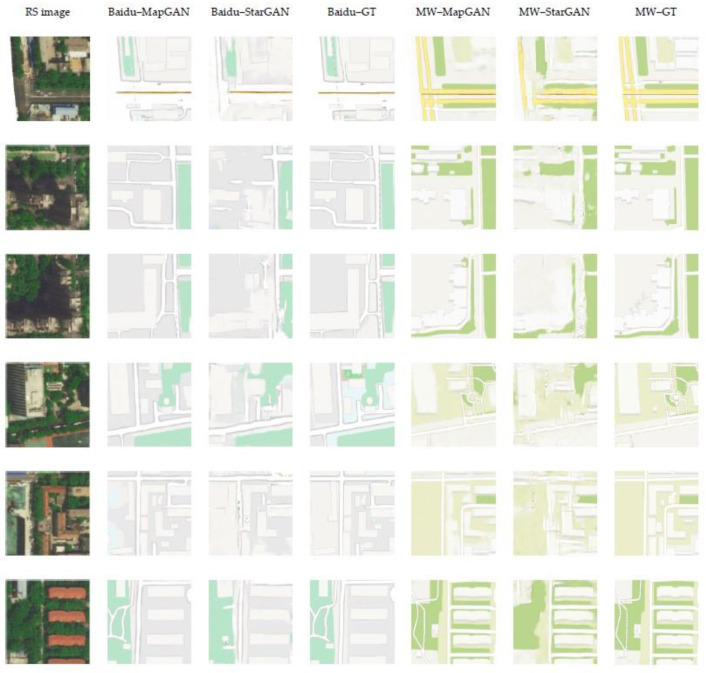
Comparison results of the Baidu and Map World electronic maps generated by MapGAN and StarGAN in the one-to-many map generation experiment. The images from left to right are remote sensing images, the Baidu maps generated by MapGAN and StarGAN, the real Baidu maps, the Map World maps generated by MapGAN and StarGAN, and the real Map World maps.

We compared the Baidu and Map World electronic maps generated by MapGAN and StarGAN. Note that the reason why we chose the unsupervised StarGAN as the comparison model is because almost all the one-to-many domain adversarial generation models are unsupervised types, and StarGAN is the most suitable one. The results are shown in Figure 11. We can infer that both StarGAN and MapGAN learned the expression characteristics of two types of maps and can map the elements in remote sensing images into two different types of electronic map styles. However, the ability to recognize features of StarGAN is relatively poor, which leads to the problem of blurred boundaries in the generated electronic map. StarGAN learned the correct colors of woodlands and highways in electronic maps but did not learn to normalize them, thereby affecting the continuity of roads and aesthetics in electronic maps. In contrast, although MapGAN also has problems such as blurring of generated residential blocks, it has better visual effects for map generation in general. 

#### 4.6.2. Quantitative Evaluation

Table 3 shows the evaluation results of the Baidu and Map World electronic maps generated by MapGAN and StarGAN over the entire test set under the five types of evaluation indicators. We separately judged the feature differences between the generated two types of electronic maps and real maps. Note that MapGAN’s pixel-level translation accuracy is higher than the one-to-one domain map generation task outlined in Section 4.5. We assume that this is because the map tiles in this experiment have a higher level of detail and there are no large numbers of scattered residential areas and roads. The evaluation results of these two models on the Baidu map are better than the Map World map, partly because the structure of the Baidu map is relatively simple. The evaluation results also show that MapGAN’s evaluation results are far superior to StarGAN on each type of generated electronic map.

#### 4.6.3. Ablation Study

**Figure 12:**
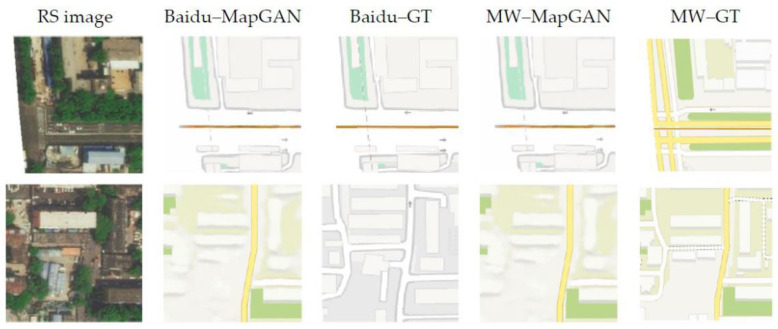
Comparison results of the Baidu and Map World maps generated by MapGAN before and after the removal of the classifier in the one-to-many map generation experiment. The images from left to right are remote sensing images, the Baidu maps generated by MapGAN, the real Baidu maps, the Map World maps generated by MapGAN, and the real Map World maps.

We used a classifier to help the model generate multiple maps of the correct type when training the model. To test the influence of the classifier on MapGAN, we removed it from the model and trained it again. The model test results are shown in Figure 12. We find that the two maps generated by the model belong to the same type and are the same. The specific generation type depends on the model initialization method and the training sequence of the samples. This is because the model now only needs to generate the electronic map to cheat the discriminator and does not need to satisfy the diversity requirement. The results in the figure are derived from two separately trained models. The type generated in the first line is the Baidu map, and the second line is the Map World map.

## 5. Conclusions

The research results in this paper mainly propose a MapGAN model that can generate kinds of realistic electronic maps based on remote sensing images and render matrices at the same time. The model uses render matrices to control the generation and color rendering of special feature elements. We also used a classifier and map classification loss term to give the model the ability to generate multiple types of maps. Experiments show that each type of electronic map generated by MapGAN exceeds the existing image translation models under many evaluation indicators including pixel-level translation accuracy, Kernel MMD, FID, mode score, and inception score. However, due to the difficulty of obtaining trainable datasets, this study only tested the model performance when generating two types of maps, and it was not known how many electronic maps a model could simultaneously generate at most. If an electronic map with many features needs to refer to third-party database information for color rendering, multiple render matrices need to be created, but considering that most of the elements of the render matrix in the model input are 0, there exists the problem of memory resource waste to some extent, which slows down the training speed of the model. In the next step, creating more trainable datasets to test our model and finding a new encoding method for the render matrix will be the direction and focus of research.

## Figures and Tables

**Table 1:** L2 loss value of feature matrix of remote sensing image and electronic map in VGG-19 part network layer.

Relu1_1	Relu1_2	Relu2_1	Relu2_2	Relu3_1	Relu3_2	Relu3_3	Relu3_4
2.4823	4.9174	6.8040	14.8359	15.5887	18.1074	16.4904	37.4131

**Table 2:** Evaluation results of Google Maps generated by MapGAN and some other image translation models in the one-to-one map generation experiment under five different indicators. The first column in the table represents the image translation models used in the test, and the first row represents the indicators used for evaluation.

	Pixel-Level Translation Accuracy	Kernel MMD	Inception Score	Mode Score	FID
BicycleGAN	0.554	0.391	1.630	1.391	0.098
MSGAN	0.613	0.357	1.610	1.474	0.076
Pix2pix	0.538	0.427	1.413	1.233	0.105
MapGAN	0.761	0.283	1.672	1.509	0.081
MapGAN-Render Matrix	0.682	0.325	1.644	1.452	0.084

**Table 3:** Evaluation results of the Baidu and Map World (MW) electronic maps generated by MapGAN and StarGAN in the one-to-many map generation experiment under five different indicators. From top to bottom are the evaluation results of the Baidu maps generated by MapGAN, the Map World maps generated by MapGAN, the Baidu maps generated by StarGAN, and the Map World maps generated by StarGAN.

	Pixel-Level Translation Accuracy	Kernel MMD	Inception Score	Mode Score	FID
Baidu–MapGAN	0.861	0.270	2.142	2.214	0.034
MW–MapGAN	0.773	0.313	2.367	2.130	0.056
Baidu–StarGAN	0.566	0.489	1.211	0.955	0.099
MW–StarGAN	0.514	0.492	1.135	0.817	0.102
